# Endothelial to Mesenchymal Transition (EndoMT) in the Pathogenesis of Human Fibrotic Diseases

**DOI:** 10.3390/jcm5040045

**Published:** 2016-04-11

**Authors:** Sonsoles Piera-Velazquez, Fabian A. Mendoza, Sergio A. Jimenez

**Affiliations:** 1 Jefferson Institute of Molecular Medicine, Department of Dermatology and Cutaneous Biology, Thomas Jefferson University, 233 S. 10th Street, Suite 509 BLSB, Philadelphia, PA 19107, USA; maria.piera-velazquez@jefferson.edu; 2 Rheumatology Division, Department of Medicine, Thomas Jefferson University, 233 S. 10th Street, Suite 509 BLSB, Philadelphia, PA 19107, USA; Fabian.A.MendozaBallesteros@jefferson.edu

**Keywords:** Endothelial Mesenchymal Transition, EndoMT, fibrosis, fibrotic diseases, systemic sclerosis, idiopathic pulmonary fibrosis, endothelial cell, myofibroblast, collagen, extracellular matrix, transforming growth factor-β

## Abstract

Fibrotic diseases encompass a wide spectrum of clinical entities including systemic fibrotic diseases such as systemic sclerosis, sclerodermatous graft *versus* host disease, nephrogenic systemic fibrosis, and IgG_4_-associated sclerosing disease, as well as numerous organ-specific disorders including radiation-induced fibrosis, and cardiac, pulmonary, liver, and kidney fibrosis. Although their causative mechanisms are quite diverse, these diseases share the common feature of an uncontrolled and progressive accumulation of fibrous tissue macromolecules in affected organs leading to their dysfunction and ultimate failure. The pathogenesis of fibrotic diseases is complex and despite extensive investigation has remained elusive. Numerous studies have identified myofibroblasts as the cells responsible for the establishment and progression of the fibrotic process. Tissue myofibroblasts in fibrotic diseases originate from several sources including quiescent tissue fibroblasts, circulating CD34+ fibrocytes, and the phenotypic conversion of various cell types including epithelial and endothelial cells into activated myofibroblasts. However, the role of the phenotypic transition of endothelial cells into mesenchymal cells (Endothelial to Mesenchymal Transition or EndoMT) in the pathogenesis of fibrotic disorders has not been fully elucidated. Here, we review the evidence supporting EndoMT’s contribution to human fibrotic disease pathogenesis.

## 1. Human Fibrotic Disorders

Fibrotic diseases encompass a wide spectrum of entities characterized by the progressive and uncontrolled accumulation of exaggerated amounts of fibrotic tissue in various organs. The resulting fibrosis disrupts the normal architecture of the affected organs, leading to their progressive dysfunction and eventually to their functional failure [1,2,3]. The most common fibrotic disorders include systemic diseases such as systemic sclerosis (SSc) [4,5,6], nephrogenic systemic fibrosis (NSF) [7,8], sclerodermatous graft *versus* host disease (GVHD) [9], and IgG_4_-associated sclerosing disease [10,11], as well as chemotherapeutic and radiation-induced fibrotic diseases [12,13], and organ-specific disorders including cardiac [14], pulmonary [15], liver [16], and kidney fibrosis [17]. Furthermore, there are other common fibrotic disorders caused by exposure to certain dusts, fibers, or chemicals such as pneumonoconiosis [18], asbestosis [19], or aristolochic acid nephropathy [20], and others that are caused by certain pathogens such as Schistosomiasis [21] and Chagas Disease [22]. The most common fibrotic diseases are listed in Table 1.

Fibrotic disorders collectively affect a very large number of individuals, and owing to the current lack of effective therapeutic approaches, result in very high morbidity and mortality rates. Indeed, it has been estimated that the mortality caused by fibrotic diseases in the Western developed countries is as high as 45% of the total mortality [3]. Although the etiology of fibrotic diseases varies widely, these conditions display as a common feature an elevated expression of genes encoding various collagens (types I, III, V, and VI) and other extracellular matrix (ECM) proteins such as fibronectin and cartilage oligomeric matrix protein (COMP) [23,24,25]. The increased expression of these genes is accompanied by a concomitant reduction in the activity of ECM degrading enzymes caused by the heightened production of Tissue Inhibitors of Metalloproteinases also known as TIMPS [26]. The combined effects of these biochemical and molecular alterations result in the exaggerated and uncontrolled accumulation of ECM macromolecules in the affected tissues.

Given the wide variety of affected organs, the chronic nature of the fibrotic processes, the lack of effective antifibrotic therapies, and the large number of individuals suffering their devastating effects, fibrotic diseases pose a serious public health problem representing an enormous challenge to health services worldwide and causing a serious economic burden to society. Despite the remarkable detrimental consequences of the fibrotic disorders for human health, there are very few approved therapeutic agents for these conditions. However, the recent elucidation of important alterations in crucial regulatory pathways involved in the fibrotic process and the identification of the central role of activated myofibroblasts in the production and abnormal deposition of ECM in this process provide a sound basis for the search and eventual identification of novel and effective means of therapy for these devastating diseases [27,28,29].

## 2. Myofibroblasts: The Effector Cells Responsible for Tissue Fibrosis

Numerous recent studies have demonstrated that myofibroblasts are the cells ultimately responsible for the severe fibrotic process in the fibrotic disorders [30,31,32,33]. Myofibroblasts comprise a unique population of mesenchymal cells that express α-smooth muscle actin (α-SMA) and exhibit a marked profibrotic cellular phenotype with increased production of fibrillar collagens (types I, III) and other ECM macromolecules, as well as an increase in TIMP production causing a reduction of the activity of ECM-degradative enzymes [26,34,35]. Furthermore, owing to their intrinsic contractile properties and their ability to establish rigid macromolecular stress fiber-like microfilament bundles *in vivo*, myofibroblasts induce changes in the biomechanical properties of the affected tissues causing a progressive increase in tissue stiffness, a newly recognized extremely potent profibrotic stimulus [36,37].

Given the crucial role of myofibroblasts in the pathogenesis of systemic and organ-specific fibrotic disorders including SSc and Idiopathic Pulmonary Fibrosis (IPF), there has been extensive investigation aimed at the precise identification of their cellular origin [38,39,40,41]. These studies have shown that myofibroblasts can originate from various sources including expansion and activation of quiescent resident tissue fibroblasts [42], migration and tissue accumulation of bone marrow-derived CD34+ fibrocytes [43,44], or from the phenotypic transition of epithelial cells, a process known as epithelial mesenchymal transition or EMT, or other cell types such as pericytes, adipocytes, or macrophages into activated myofibroblasts [45,46,47]. More recent studies, however, have demonstrated that another source of activated myofibroblasts in fibrotic diseases are endothelial cells (EC) that have acquired a mesenchymal phenotype through a process known as endothelial to mesenchymal transition (EndoMT). This hypothesis, first proposed by Karasek [48], has been pursued intensively and numerous subsequent studies have examined the possible role of EndoMT in the pathogenesis of these disorders. During EndoMT EC become delaminated and detach from the endothelial layer, change their morphologic characteristics becoming elongated and fusiform, lose their specific EC molecular markers such as CD31/PECAM-1, von Willebrand Factor (vWF), and VE-cadherin, and initiate the expression of mesenchymal cell products including α-SMA, vimentin, and type I collagen [34,35]. The occurrence of EndoMT was initially described during embryonic development and it was shown to play a crucial role in the formation of the mesenchymal cushion during the development of cardiac valves [49,50]. Numerous subsequent studies have described EndoMT in various animal models of experimentally-induced fibrotic diseases, including cardiac, pulmonary, renal, and radiation induced fibrosis [51,52,53,54], as well as in various human disorders associated with tissue fibrosis [55,56,57,58,59,60,61,62,63,64,65,66]. Despite these extensive observations, however, the possibility that EndoMT participates in the development or progression of human fibrotic diseases has been considered controversial [67]. In this regard, it is important to emphasize that although myofibroblasts are the key cellular element responsible for the fibrotic process, it is likely that the transition from EC into myofibroblasts may not need to proceed through complete transdifferentiation and that a partial transition process may be sufficient for initiation or progression of pathologic fibrogenesis as discussed recently by Welch-Reardon *et al.* [68]. Indeed, there is strong experimental evidence from several EMT studies demonstrating that partial EMT transition without a complete progression of epithelial cells into myofibroblasts may participate in pathologic fibrogenesis [69,70]. However, as similar studies have not been performed in EndoMT the issue of whether partial EndoMT participates in the fibrotic process still remains unsolved.

In the following sections, we will review the available evidence that supports a role for EndoMT in the pathogenesis of various human fibrotic disorders. First, we will review studies of the molecular mechanisms of EndoMT emphasizing various pathways known to participate in the pathogenesis of tissue fibrosis, then, we will briefly discuss the occurrence of EndoMT in various experimentally-induced animal models of tissue fibrosis, and finally, we will review the occurrence of EndoMT in cultured human EC and in tissues from patients with various human fibrotic disorders.

## 3. Pathways Involved in Tissue Fibrosis Participate in the Molecular Mechanisms of EndoMT

**Figure 1:**
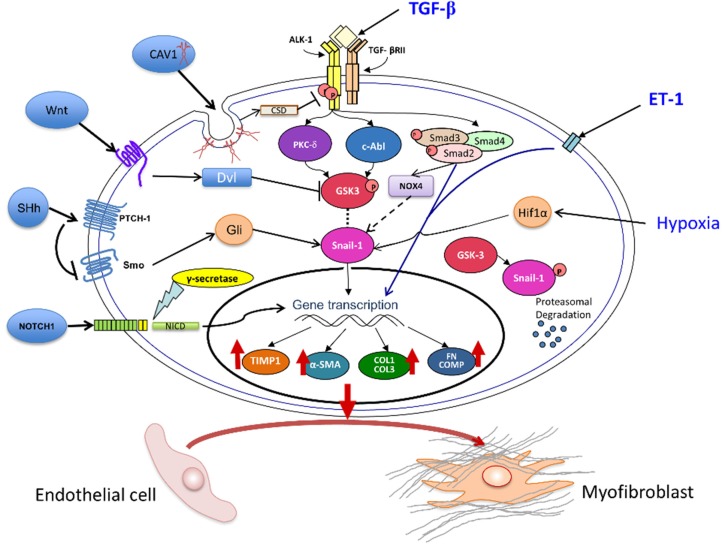
Molecular Mechanisms of EndoMT. The diagram shows the TGF-β, ET-1, NOTCH, CAV-1, Wnt, NOX4, and HIF-1α pathways that may participate in the EndoMT process. The most important pathway is initiated following TGF-β-binding and subsequent activation of Smad-dependent and Smad-independent TGF-β intracellular signaling. TGF-β causes a direct stimulation of NOX4 expression that results in Snail1 mediated EndoMT. ET-1 induces a synergistic stimulation of TGF-β-induced EndoMT involving the canonical Smad pathways. Hypoxia also induces EndoMT through the effects of Hif1α activation of Snail1. Snail1 has emerged as a crucial regulatory molecule in EndoMT and its levels are modulated by GSK3-mediated phosphorylation as phosphorylated Snail1 undergoes proteasomal degradation. Cav1 exerts an inhibitory effect owing to the internalization of TGF-β receptors and their subsequent degradation. Morphogen pathways including Wnt, Sonic Hh, and NOTCH also may modulate EndoMT. The ultimate effect of these complex intracellular signaling events is the activation of a mesenchymal cell specific transcriptional gene regulation program leading to the increased production of various myofibroblast-specific and profibrotic macromolecules including α-SMA, COL1, COL3, FN, COMP, and the MMP-inhibitor TIMP. These events are accompanied by the repression of EC-specific gene products such as CD31/PECAM-1, VE-cadherin, and von Willebrand Factor (not shown in the diagram) resulting in the phenotypic conversion of EC into myofibroblasts, the cells ultimately responsible for the fibrotic process.

In contrast with the extensive studies on the molecular events and pathways involved in EMT [71], the mechanisms regulating the EndoMT process have not been fully elucidated, and only a few studies have examined the molecular changes and the regulatory events occurring in EC during their mesenchymal transdifferentiation or phenotypic conversion into activated myofibroblasts. Some of the complex pathways involved in EndoMT-mediated generation of myofibroblasts from EC are diagrammatically shown in Figure 1. In the following sections the recent evidence demonstrating the crucial role of TGF-β and other signaling pathways in the initiation and establishment of EndoMT will be briefly reviewed.

### 3.1. The Transforming Growth Factor-β (TGF-β) Pathway

The TGF-β family of proteins comprises several pleiotropic growth factors that play crucial roles in numerous physiological processes including embryogenesis, cellular development and differentiation, immunologic system development, inflammatory response functions, and wound repair [71,72]. Owing to their potent profibrotic effects these molecules have also been implicated in the pathogenesis of various fibrotic human diseases [73,74,75,76,77,78]. TGF-β1, the most well studied member of this protein family is the most potent profibrogenic growth factor identified to date. Several mechanisms are involved in the profibrotic effects of TGF-β1, including the transcriptional stimulation of the expression of numerous genes involved in the fibrotic process such as various collagens and proteoglycans, fibronectin, COMP, PAI, CTGF, NOX4, and other relevant genes that play important roles in various aspects of the fibrotic process. Furthermore, numerous studies have shown that the three TGF-β isoforms are potent inducers of myofibroblasts either through activation of quiescent fibroblasts, or through the phenotypic conversion of various cell types into activated myofibroblasts [52,79,80,81].

Given the crucial role that the TGF-β family of profibrotic growth factors plays in the development of tissue fibrosis and in the pathogenesis of numerous fibrotic diseases, several studies have demonstrated that TGF-β is involved in the generation of myofibroblasts through EndoMT [79,80,81,82,83,84]. The detailed molecular events and the intracellular cascades activated by TGF-β that result in the phenotypic change of EC into mesenchymal cells are highly complex and have not been entirely elucidated. However, it has been shown that both Smad-dependent and Smad-independent pathways and numerous transcriptional regulators such as Snail1, Snail2 (or Slug), Twist, and some members of Zeb family of proteins are involved [79,80,81,82,83]. We recently examined the intracellular transduction pathways mediating TGF-β-induced EndoMT in cultured immunopurified murine lung EC [84], and made the following observations: (1) primary murine pulmonary endothelial cells undergo EndoMT in response to TGF-β with initiation of expression of α-SMA and assembly of typical intracellular α-SMA stress fibers and loss of VE-cadherin *in vitro*; (2) TGF-β induction of EndoMT was associated with a strong upregulation in the expression of the transcriptional repressor Snail1 indicating that Snail1 is directly involved in TGF-β-induced α-SMA expression; and (3) induction of α-SMA expression involved the c-Abl kinase and protein kinase C-δ (PKC-δ), as specific inhibition of their kinase activities with imatinib mesylate and rottlerin, respectively, or by knockdown of their corresponding transcripts with specific siRNA abrogated the marked increase in TGF-β induced α-SMA and Snail1 expression and protein levels. These observations confirmed the potent induction of EndoMT by TGF-β1 *in vitro* [84]. The relevant role of TGF-β signaling in the induction of EndoMT has been further confirmed in several *in vivo* studies. The study of Cooley *et al.* demonstrated that a TGF-β-neutralizing antibody decreased EndoMT and resulted in a profound reduction in neo-intima formation in a mouse model of interpositional vein grafts [61]. In another study, Xavier *et al.* generated heterozygous mice with endothelium-specific knock-out of the TGF-receptor II (TβRII) gene and demonstrated that the resulting partial TβRII deletion abrogated EndoMT and reduced tubulointerstitial kidney fibrosis in two murine models of renal fibrosis [85]. The role of TGF-β in EndoMT was further emphasized in a study demonstrating that inhibition of TGF-β receptor heterodimer formation by knockdown of either integrin β1 or dipeptidyl peptidase-4 in cultured EC resulted in suppression of EndoMT and was associated with less severe kidney fibrosis [86].

### 3.2. Role of Notch and Hedgehog Signaling Pathways

The morphogens Notch and Hedgehog play crucial roles during embryonic development. Although in the adult these pathways are very highly regulated, their aberrant activation may lead to various pathological consequences, including the development of fibrotic diseases [87,88,89]. The role of Notch signaling in EndoMT was first described in human microvascular EC and human umbilical vein EC [90]. Although the mechanisms involved have not been fully elucidated, recent studies have shown that in human microvascular EC, Notch, and TGF-β synergistically stimulate Snail expression and upregulate numerous genes by recruiting Smad3 to Smad binding sites in their corresponding gene promoter regulatory elements [91,92,93]. However, the potential role of Notch-induced EndoMT in human fibrotic diseases has not been studied in detail.

Recently, the Sonic Hedgehog (SHh) pathway, another important morphogen, has also been shown to participate in the pathogenesis of various fibrotic disorders [94,95,96,97,98]. An extensive immunofluorescence analysis for SHh epitopes in affected SSc skin showed intense staining in dermal fibroblasts and EC [98]. Other results from this study supported the potential role of SHh in tissue fibrosis as it was shown that TGF-β increased its expression and that SHh induced strong stimulation of fibroblast to myofibroblast transition in normal human dermal fibroblasts with a potency comparable to that of TGF-β [98]. Despite these observations, however, the possibility that SHh may be involved in EndoMT has not been examined.

### 3.3. Wnt Pathway Effects on EndoMT

The Wnt proteins comprise a multigene family of secreted glycoproteins that play crucial roles during embryogenesis signaling through canonical and non-canonical pathways [99,100]. Numerous recent studies have shown the participation of Wnt proteins in tissue fibrosis. The Wnt/β-catenin pathway is involved in the activation of numerous profibrotic steps inducing myofibroblast differentiation via Smad-dependent autocrine TGF-β signaling, thus, promoting pathologic fibrogenesis [101,102,103,104]. Two recent studies demonstrated that induction of canonical Wnt signaling resulted in EndoMT in cultured EC [105,106]. One study showed that a significant proportion of α-SMA positive myofibroblasts displaying β-catenin dependent Wnt signaling were derived from myocardial EC following experimentally-induced myocardial infarction [105]. The other study showed that human renal glomerular EC cultured in a high glucose medium underwent EndoMT in a process that involves β-catenin [106]. In contrast, another study showed that the Wnt inhibitor Dkk-1 enhanced EndoMT in aortic EC [107]. These apparently contradictory results indicate that further study is needed to conclusively determine the precise role of Wnt and Wnt-related proteins on EndoMT.

### 3.4. Caveolin-1 (CAV1) Modulation of EndoMT

CAV1, the main protein component of caveolae plays an important role in the internalization, trafficking and degradation of TGF-β receptors and, therefore, is involved in the regulation of TGF-β signaling and TGF-β-mediated fibrotic responses [108,109,110]. Studies in *Cav1* knock out mice (*Cav1*−/−) showed that these mice exhibited extensive skin and lung fibrosis [111]. Subsequent investigation demonstrated that CAV1 protein and gene expression were markedly decreased in affected tissues from patients with SSc and idiopathic pulmonary fibrosis [111,112,113], and that restoration of CAV1 functional domains corrected the profibrotic phenotype [114]. These studies, however, did not examine whether CAV1 was involved in EndoMT. To address this point, we examined the role of CAV1 in EndoMT employing immunopurified EC isolated from lungs of *Cav1*−/− mice [115]. The results demonstrated that EndoMT occurred spontaneously in *Cav1*−/−mice *in vivo* and suggested that CAV1 deficiency participates in the development and progression of tissue fibrosis and fibrotic diseases through the establishment of EndoMT.

### 3.5. The Role of Endothelin-1 (ET-1) in EndoMT

The important role of ET-1, the most potent endogenous vasoconstrictor polypeptide identified to date, in the regulation of multiple vascular functions and its participation in various human diseases including Primary and Secondary Pulmonary Arterial Hypertension (PAH) has been well recognized. However, numerous recent studies have shown that ET-1 also displays strong profibrotic effects and it is involved in various aspects of the fibrotic process [116,117]. Recent studies have examined whether ET-1 is capable of inducing EndoMT. One of the earliest demonstrations of the prominent role of ET-1 in the development of EndoMT was obtained in experimentally-induced diabetes in mice with normal ET-1 levels and in ET-1 knockout mice [118]. These studies showed that EC-derived ET-1 promotes cardiac fibrosis and heart failure in diabetic hearts through stimulation of EndoMT. In recent studies from our group employing murine lung EC, we found that although ET-1 was not capable of inducing EndoMT by itself it exerted potent synergistic stimulation of TGF-β effects on EndoMT. The contribution of ET-1 was confirmed by treatment of the cells with the ET-1-specific inhibitor, Bosentan, which abrogated the synergistic stimulation of TGF-β1-induced EndoMT by ET-1 [119]. In contrast to our results, another study employing immunopurified CD31 dermal EC from SSc patients and from normal individuals showed that either TGF-β or ET-1 were able to induce EndoMT in normal and SSc-EC and that these effects involved the Smad pathway. The important contribution of ET-1 to these effects was confirmed as they were blocked by the specific ET-1 receptor antagonist, macitentan [120]. The results of the studies with human EC demonstrate that ET-1 either by itself or in combination with TGF-β is capable of generating activated tissue myofibroblasts through EndoMT. These results combined with those from our study employing murine lung EC provide a novel mechanism for ET-1 participation in the development of tissue fibrotic reactions.

### 3.6. Involvement of Hypoxia in EndoMT

Numerous recent studies have demonstrated an important role of hypoxia in the development or progression of various fibrotic diseases including SSc, kidney, and cardiac fibrosis [121,122,123,124]. The transcription factor HIF-1α is the key regulatory molecule responsible for the cellular and molecular responses to hypoxia [121]. There are several mechanisms involved in HIF-1α-induced fibrosis including the activation of profibrotic genes [125] and the synergistic interaction of HIF-1α with profibrotic growth factors such as TGF-β and VEGF [126]. Furthermore, an extensive study employing genetic inactivation of HIF-1α in murine renal proximal tubule epithelial cells showed that HIF-1α enhanced EMT *in vitro* and *in vivo* [127]. However, the possibility that hypoxia and HIF-1α may play a role in fibrotic diseases through induction of EndoMT has just begun to be explored. Indeed, one recent study showed that hypoxia induced EndoMT in human coronary EC, an effect mediated by a potent activation of Snail, and suggested that these changes may ultimately lead to development of cardiac fibrosis [128] and another study showed that HIF-1α induced EndoMT during the development of radiation-induced pulmonary fibrosis [129].

### 3.7. Role of NOX4 and Oxidative Stress in EndoMT

One novel pathway that has been recently recognized as a potentially important participant in the pathogenesis of fibrotic processes through reciprocal interactions with TGF-β involves reactive oxygen species (ROS) [130,131]. Although ROS are produced by normal fibroblasts and are essential for numerous important intracellular reactions, several studies have implicated the generation of deleterious ROS in the pathogenesis of various fibrotic disorders including SSc, IPF, and liver fibrosis [132,133,134].

Although there are multiple sources of intracellular ROS, extensive studies have shown that most ROS production derives from the activation of the nicotinamide adenine dinucleotide phosphate (NADPH) oxidase system. NOX4 is one of seven NADPH isoforms, however, unlike other members of the NOX family, it is constitutively active and the regulation of its effects occurs mainly at the expression level. The role of NOX4 as an important downstream mediator of TGF-β-induced myofibroblast generation, its contribution to the potent TGF-β profibrotic effects, and its participation in the pathogenesis of tissue fibrosis in various fibrotic disorders such as IPF and liver fibrosis have been recently demonstrated [135,136,137,138,139,140,141,142,143,144]. In recent studies from our group, we found increased levels of NOX4 in affected SSc skin and increased expression of NOX4 transcripts in cultured SSc dermal fibroblasts. We also confirmed results of previous studies showing that TGF-β caused a potent stimulation of NOX4 gene expression and of NOX4 protein levels, thus demonstrating that NOX4 was involved in the profibrotic effects of TGF-β1 employing a potent and highly selective NOX4 inhibitor [144].

### 3.8. Role of MicroRNA in EndoMT

MicroRNAs (miRNAs) are small non-coding RNA that modulate the expression of a large number of protein coding genes at the post-transcriptional level [145]. Recent interest has been devoted to elucidating their participation in tissue fibrosis and numerous miRNA have been shown to be involved in the fibrotic process exerting either profibrotic or antifibrotic effects. These effects are mediated by their ability to target a large number of translated mRNAs coded by genes involved in ECM structure, function and homeostasis, such as those encoding collagens, matrix metalloproteinases, Smad signaling proteins, and even TGF-β itself [146,147,148]. Furthermore, numerous miRNA have been shown to play important roles in the pathogenesis of SSc and other cutaneous fibrotic disorders [149,150], IPF [151], and cardiac, liver, and kidney fibrosis [152,153,154]. However, the possibility that the profibrotic or antifibrotic effects of specific miRNAs may be mediated through modulation of EndoMT has not been studied extensively, although some recent studies have described the contribution of various miRNA to the EndoMT process. Among these studies are the identification of the effects of miRNAs 125b and 126 on the development of EndoMT [155,156,157], the demonstration that TGF-β-induced EndoMT is partially mediated through miRNA21 [158,159], and that miR-155 is significantly upregulated in EndoMT and is a potent inhibitor of TGF-β induced EndoMT, an effect mediated through the modulation of RhoA signaling [160]. In contrast, a very recent study showed that miR-31 is a positive modulator of TGF-β-induced EndoMT and that it was required for the expression of α-SMA and other EndoMT molecular markers following TGF-β-treatment [161]. Several other miRNA have also been shown to modulate EndoMT including let-7 [162,163] and miRNA 29s [164]. Given the remarkable regulatory effects of miRNAs on the expression of a large number of relevant mRNA transcripts, it is expected that the exploration of miRNA contribution to the phenotypic conversion of EC into mesenchymal cells will rapidly expand and will yield very valuable information both to unravel the complex mechanisms involved in the EndoMT process, as well as to allow the identification of potential therapeutic targets for the fibrotic disorders.

## 4. EndoMT in Animal Models of Tissue Fibrosis

### 4.1. Cardiac Fibrosis

The pioneering studies of Zeisberg *et al.* [51] clearly demonstrated the occurrence of EndoMT during the development of experimentally-induced cardiac fibrosis. In this study, cardiac fibrosis was induced in mice following aortic banding and the development of EndoMT was assessed employing endothelial cell lineage analysis in transgenic mice. The quantitative analysis of the proportion of EndoMT-derived fibroblasts present in myocardial fibrosis tissues showed that 27% to 35% of fibroblasts originated from EC [51]. Another study demonstrated the contribution of EndoMT to the development of cardiac fibrosis induced by isoproterenol administration to rats [165]. Other studies have confirmed these observations and, collectively, have suggested that EndoMT represents an important contributor to cardiac fibrosis [52,118]. However, in contrast with these various reports, two recent studies utilizing multiple independent murine lines and a fibroblast-specific fluorescent reporter marker in a model of cardiac pressure overload indicated that the fibroblasts responsible for cardiac fibrosis were derived from resident epicardial and interstitial fibroblast pools and not from EndoMT, thus, raising some controversy about the role of EndoMT in the development of cardiac fibrosis and other fibrotic disorders [67].

### 4.2. Renal Fibrosis

Li and Bertram [54] recently reviewed the experimental evidence showing that EndoMT is a novel pathway leading to fibrotic development in diabetic nephropathy and other animal models of kidney fibrosis. Several other studies have examined the development of EndoMT in various animal models of kidney fibrosis. The study by Zeisberg *et al.* [53] examined the contribution of EndoMT to the development of renal fibrosis in three different animal models of end-stage kidney disease, the unilateral ureteral obstruction, the streptozotocin-induced diabetic nephropathy, and the COL4A3 knockout murine model of Alport’s syndrome. The results showed numerous myofibroblasts co-expressing EC and mesenchymal cell markers in the three models of kidney fibrosis. They also performed studies with an endothelial lineage-traceable mouse strain. These studies indicated that 30% to 50% of myofibroblasts in the fibrotic kidneys were of endothelial cell origin [53]. In another study Li *et al.* [166] employed a Tie2Cre/Lox P-EGFP mouse strain that allows the expression of the green fluorescent protein marker in any cell of endothelial origin even after the cell has undergone a phenotypic change. They induced diabetic nephropathy in these mice employing streptozotocin and demonstrated the presence of numerous myofibroblasts of endothelial origin in the diabetic kidneys. A quantitative assessment of the proportion of endothelial-derived myofibroblasts employing confocal microscopy indicated that 10% to 23% of myofibroblasts in the fibrotic tissue were labeled with the endothelial-specific marker and were, therefore, of endothelial origin [166]. These studies were recently validated by an extensive study by LeBleu *et al.* [57] employing various genetically engineered mouse strains and endothelial cell lineage tracing. The results showed that approximately 10%–15% of activated myofibroblasts in the fibrotic kidneys originated from EC through EndoMT and conclusively demonstrated the occurrence of EndoMT during the development of kidney fibrosis as discussed recently [54,167].

### 4.3. Pulmonary Fibrosis and Pulmonary Arterial Hypertension

The role of EndoMT in experimentally induced pulmonary fibrosis and pulmonary arterial hypertension was examined in several recent studies. The pioneering studies of Hashimoto *et al.* [168] evaluated EndoMT as a source of interstitial fibroblasts in bleomycin-induced lung fibrosis. Following endotracheal injection of bleomycin, the areas of fibrotic involvement were shown to contain large numbers of myofibroblasts of endothelial origin. Furthermore, lung fibroblasts cultured from either saline injected control mice or from mice that received bleomycin injections showed that about 15% to 20% of lung fibroblasts in the cultures from bleomycin-treated mice co-expressed endothelial and mesenchymal cell markers demonstrating their EndoMT origin. Another study examined the occurrence of EndoMT in PAH induced in mice following exposure to hypoxia and treatment with the potent VEGF receptor inhibitor, SU5416 [65]. The results showed numerous pulmonary arterioles displaying colocalization of vWF and α-SMA indicative of the occurrence of EndoMT *in vivo* in this animal model.

## 5. *In Vitro* Studies of EndoMT in Human Endothelial Cells

In contrast with the extensive evidence from animal models demonstrating the occurrence of EndoMT in the development of experimentally-induced tissue fibrosis there are only few studies that have provided evidence indicative of the occurrence of EndoMT in human fibrotic disorders. Most studies have been performed *in vitro* with EC isolated from affected tissues. The study of Zeisberg *et al.* [51] describing for the first time the occurrence of EndoMT in an animal model of aortic-banding induced cardiac fibrosis included some *in vitro* studies with isolated human coronary artery EC. These studies showed that, following TGF-β1 treatment, the cells became spindly shaped and lost their EC molecular markers and acquired the ability to produce various fibroblast-specific macromolecules including vimentin and type I procollagen. Several other studies have examined the occurrence of EndoMT in cultured human EC of various types. A study by Kitao *et al.* [62] examined the effects of TGF-β1 *in vitro* in cultured human dermal microvascular EC and demonstrated the induction of a mesenchymal cell phenotype by TGF-β1 with a change to a spindle cell morphology in monolayer culture and initiation of expression of α-SMA and COL1A. In another study Rieder *et al.* described the phenotypic conversion of human microvascular intestinal EC into mesenchymal cells following exposure to a combination of proinflammatory cytokines (TGF-β, TNF-α and IL-1β) *in vitro* and suggested that intestinal EC exposed to an inflammatory environment may participate in the intestinal fibrotic process which accompanies intestinal inflammatory diseases *in vitro* [59]. Chrobak *et al.* examined the effects of interferons α and γ on human dermal microvascular EC and demonstrated that interferon γ induced morphological and molecular/gene expression changes in these cells consistent with their transdifferentiation into profibrotic myofibroblasts [169]. Recently, the mechanisms of radiation-induced EndoMT were investigated in human pulmonary artery endothelial cells (HPAEC) *in vitro* [129]. The results showed that radiation exposure of these cells resulted in a radiation dose and length of exposure-dependent increase in the expression of various fibroblast-specific markers with a concomitant decrease in EC-specific markers indicating that radiation triggered EndoMT in these cells. Subsequently, it was shown that the effects were mediated by the canonical SMAD signaling cascade triggered by TGF-β receptor activation [129]. The same study described EndoMT changes following hypoxic exposure including increased phosphorylation of Smad3, and increased expression of TGF-β receptor I and Snail1. These data indicated that hypoxia triggers EndoMT through HIF1α-mediated activation of TGF-β receptor I/Smad signaling [129].

## 6. EndoMT in Human Fibrotic Diseases

All the studies described in the previous section demonstrated that EC isolated from fibrotic tissues from various human fibrotic disorders were capable of undergoing EndoMT *in vitro* following exposure to TGF-β1 or to other cytokines or inducing agents. In contrast, there are few studies that have demonstrated the occurrence of EndoMT directly *in vivo*, although some recent publications have described results demonstrating the presence of EC expressing simultaneously EC molecular markers such as, for example, CD31/PECAM-1 or von Willebrand Factor and proteins such as α-SMA, or type I and III interstitial collagens that are specific for myofibroblastic mesenchymal cells. Most of these studies identified cells co-expressing both types of molecular markers in the affected tissues employing immunofluorescence microscopy studies with confocal microscopy. These studies are summarized in Table 2 and the results will be discussed in the following sections.

### 6.1 Idiopathic Pulmonary Fibrosis and Pulmonary Arterial Hypertension

Despite the clinical importance and the demonstration of morphological and molecular alterations indicative of EndoMT during experimentally-induced pulmonary fibrosis [168], there are no reports describing EndoMT in IPF or in other forms of secondary pulmonary fibrosis. However, some studies have examined the role of EndoMT in PAH. Although PAH is not considered among the fibrotic diseases, in its chronic stages substantial sub-endothelial and adventitial perivascular fibrosis are frequently present. Furthermore, PAH is often a manifestation of typical fibrotic disorders such as SSc and IPF, thus several studies have described results supporting a role of EndoMT in the development of the fibrotic alterations present in the pulmonary vasculature in PAH. Arciniegas *et al.* [172] were among the first investigators to suggest a role of EndoMT in chronic PAH. Following this novel suggestion, two more recent studies implicated EndoMT in the pathogenesis of PAH, one study examined Primary PAH [65] and the other studied PAH secondary to SSc [66]. In the Primary PAH study, Ranchoux *et al.* [65] employed transmission electron microscopy and correlative light and electron microscopy. The results provided unequivocal ultrastructural-level evidence of ongoing EndoMT in lung tissue samples from patients with primary PAH. Indeed, it was shown that typical EC identified by the presence of Weibel-Palade bodies acquired expression of the myofibroblast-specific marker α-SMA, as well as displayed invaginations into the neointima of the abnormal pulmonary arterioles. In the study of SSc-associated PAH, Good *et al.* [66], assessed EndoMT in the pulmonary arterioles in SSc lung tissues. Examination of the cellular phenotype in intimal and plexiform lesions from PAH lungs showed the unambiguous co-expression of endothelial and mesenchymal markers in up to 4% of pulmonary arterioles in the lungs of patients with SSc-associated PAH. Furthermore, the protein and mRNA expression patterns of the tissues confirmed the contribution of EndoMT to SSc-associated PAH pathology. The novel observations described in these two studies provide conclusive evidence for the occurrence of EndoMT in small and medium size arterioles of lung tissues from patients with primary and SSc-associated PAH.

### 6.2. Systemic Sclerosis

**Figure 2:**
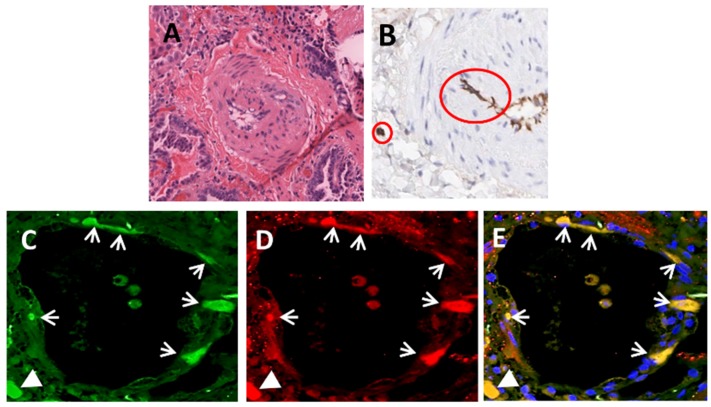
Immunohistology and confocal microscopy staining of medium-sized pulmonary arteries in SSc-associated pulmonary fibrosis lung tissues. (**A**) Histopathology of a pulmonary arteriole showing severe proliferative vasculopathy with luminal occlusion; (**B**) CD31-expressing cells (brown staining) in the subendothelial region of a small pulmonary arteriole and in the lung parenchyma (red circles). (**C**–**E**) small arteriole in affected SSc lung; (**C**) Staining for vWF (green); (**D**) Staining for α-SMA (red); (**E**) Overlay (yellow). Note numerous cells in the endothelial lining (arrows) and one cell in the subendothelial tissue (arrowhead) displaying co-expression of EC (vWF) and myofibroblast (α-SMA) molecular markers as evidenced by the yellow color in the overlay image. Adapted from Ref. [64].

We recently performed a study to examine the possibility that EndoMT is involved in the fibrotic process of SSc-associated pulmonary fibrosis [64]. Immunohistology studies of SSc lung tissues showed expression of the EC marker CD31 in mesenchymal cells embedded within the neointima of small pulmonary arteries as well as in the parenchymal fibrotic areas as illustrated in Figure 2A,B. Furthermore, co-expression of CD31 or vWF with the mesenchymal markers, collagen type I or α-SMA was demonstrated employing confocal laser microscopy in numerous EC lining the small and medium sized pulmonary arteries as illustrated for small arterioles in Figure 2C–E. These findings were not present in the small or medium sized arteries of the normal lung tissues. The results demonstrated that EC co-expressing EC-specific and myofibroblastic cell markers are present in the endothelium of small pulmonary arteries from patients with SSc-associated pulmonary fibrosis and suggest that mesenchymal cells of endothelial origin are likely to be responsible for the production and accumulation of subendothelial fibrotic tissue in the affected vessels that in turn result in their luminal obliteration. These observations were confirmed by an extensive assessment of the differences in gene expression patterns between microvascular EC isolated from normal lungs compared to microvascular EC isolated from lungs from patients with SSc-associated pulmonary fibrosis [64]. The results demonstrated a very strong expression of COL1A1 and COL3A1 in the CD31+/CD102+ purified EC from lungs from SSc patients and these values were up to 21 times and 26 times higher, respectively, than the expression of the same collagen genes in CD31+/CD102+ EC purified from the normal control lungs. The expression of FN1 and ACTA2 (α-SMA), other profibrotic genes such as TGFB1 and CTGF, and that of several EndoMT-related genes such as SNAI2 and TWIST was also substantially increased in the CD31+/CD102+ EC from the lungs of SSc patients. Thus, these results provide very strong evidence for the occurrence of EndoMT during the fibrotic process affecting the lungs in SSc-associated pulmonary fibrosis and also suggest an important role for EndoMT in the development of the vascular alterations in SSc as discussed recently [173].

### 6.3. Radiation Induced Pulmonary Fibrosis

Tissue fibrotic reactions represent the most serious complications of radiation therapy and intense investigation is being conducted to understand the intimate mechanisms involved in the devastating organ fibrosis and dysfunction induced by radiation therapy. Recent studies have examined the potential role of EndoMT in the severe radiation-induced fibrotic process. One study examined the role of EndoMT in the development of radiation-induced pulmonary fibrosis [129]. Using immunofluorescence analysis, colocalization of α-SMA and CD31 was evident throughout the tissue during the development of the fibrotic process and it was accompanied by extensive collagen accumulation in the lung parenchyma. These data suggest that radiation-induced EndoMT was involved in the development of the severe and progressive fibrotic process occurring in the irradiated lungs [129]. These observations indicate that abrogation of radiation-induced EndoMT may be an effective initial therapy for prevention of radiation-induced fibrotic processes.

### 6.4. Cardiac and Renal Fibrosis

Several studies have described evidence supporting the occurrence of EndoMT in human tissues from patients with cardiac fibrosis [170,171] and from patients with kidney fibrosis associated with diabetic kidney disease [106]. The study of Xu *et al.* [170] examined the expression of various EndoMT-associated genes in left ventricular myocardial tissues from patients undergoing cardiac transplantation for end-stage cardiac failure and demonstrated a remarkable increase in the mRNA levels for Snail (6-fold), Slug (5-fold), and Twist (10-fold). The study by Charytan *et al.* [171] performed immunofluorescence and gene expression analyses of myocardial tissues from patients with cardiac fibrosis associated with chronic kidney disease and demonstrated that about 17% of fibroblasts/myofibroblasts present in the fibrotic myocardium were EndoMT-derived. Thus, these two studies provided strong evidence indicative of the occurrence of EndoMT in the development of myocardial fibrosis. Regarding kidney fibrosis, a recent study examined the presence of glomerular cells expressing EC and mesenchymal/myofibroblast cell-specific molecules (α-SMA and CD31) in kidney biopsy tissues obtained from patients with diabetic kidney disease. The results demonstrated the presence of numerous glomerular cells co-expressing α-SMA and CD31 providing strong evidence for the occurrence of EndoMT *in vivo* during the development of diabetic kidney disease-associated kidney fibrosis [106].

### 6.5. Portal Vein Fibrosis and Other Fibrotic Disorders

The possibility that EndoMT of portal vein endothelium via TGF-β/Smad activation may be involved in portal venopathy was examined recently [62]. The results showed enhanced expression of phosphorylated Smad2 in venous endothelium of smaller portal veins in idiopathic portal hypertension associated with co-localization of EC and myofibroblast protein markers. The authors concluded that the conversion of portal vein EC into cells expressing a myofibroblastic phenotype may be responsible for exaggerated periportal-venous deposition of fibrous tissue proteins and may represent the mechanism responsible for portal venous obliteration in idiopathic portal hypertension. Other studies have also described the occurrence of EndoMT in several other pathologic conditions including vascular occlusion and neointima formation in human vein graft tissues [61], intestinal fibrosis associated with inflammatory bowel disease [59,174], and radiation-induced rectal fibrosis [63].

## 7. Concluding Remarks

The results of the numerous studies reviewed here including *in vitro* studies with human EC of various tissue origins, experimental animal models of tissue fibrosis, and analysis of tissues from patients with various fibrotic diseases certainly indicate that EndoMT plays an important role in the pathogenesis of these common and often fatal disorders. The extensive literature published about the participation of EndoMT in the fibrotic process and the demonstration of its occurrence in affected tissues from patients with SSc-associated pulmonary fibrosis and PAH, vein graft fibrosis, intestinal fibrosis, and radiation-induced organ fibrosis indicate that such a role should no longer be considered controversial but it is a reality, as discussed recently [175,176]. A critical assessment of the large number of studies reviewed here clearly indicates that elucidation of the molecular mechanisms involved in EndoMT may provide novel molecular targets and therapeutic approaches for a large number of fibrotic diseases.

## Figures and Tables

**Table 1:** The spectrum of human fibrotic diseases.

A. Systemic Fibrotic Diseases
• Systemic Sclerosis • IgG_4_-associated Tissue Fibrosis • Gd-contrast Agent-induced Nephrogenic Systemic Fibrosis • Sclerodermatous Graft *vs.* Host Disease
**B. Organ-Specific Fibrotic Diseases**
Cardiac Fibrosis
• Pressure Overload • Post-myocardial-infarction • Chagas Disease-induced fibrosis
Kidney Fibrosis
• Diabetic and Hypertensive Nephropathy • Urinary Tract Obstruction-induced Kidney Fibrosis • Inflammatory/Autoimmune-induced Kidney Fibrosis • Aristolochic acid Nephropathy • Polycystic Kidney Disease
Pulmonary Fibrosis
• Idiopathic Pulmonary Fibrosis • Silica-induced Pneumoconiosis (Silicosis) • Asbestos-induced Pulmonary Fibrosis (Asbestosis) • Chemotherapeutic Agent-induced Pulmonary Fibrosis
Liver and Portal Vein Fibrosis
• Alcoholic and Non-Alcoholic Liver Fibrosis • Hepatitis C-induced Liver Fibrosis • Primary Biliary Cirrhosis • Parasite-induced Liver Fibrosis (Schistosomiasis)
**C. Other Organ-specific Fibrotic Diseases**
• Intestinal Fibrosis • Bladder Fibrosis • Radiation-induced Fibrosis (various organs) • Peritoneal Sclerosis • Localized Scleroderma, Diffuse Fasciitis, and Keloids • Dupuytren’s Disease • Peyronie’s Disease • Myelofibrosis • Oral Submucous Fibrosis

**Table 2:** Demonstration of EndoMT in human fibrotic diseases.

Fibrotic Disease	Evidence of EndoMT in Affected Tissues		Source of Data
	Tissue Source	Method(s)	
SSc-associated Pulmonary Fibrosis	Lung transplants	Immunohistochemistry	Mendoza *et al.* [64]
		Immunofluorescence	
		Gene Expression	
Radiation-induced Pulmonary Fibrosis	Lung tissues (Surgery)	Immunofluorescence	Choi *et al.* [129]
SSc-associated Pulmonary Hypertension	Lung biopsies	Immunofluorescence	Good *et al.* [66]
Idiopathic Pulmonary Hypertension	Lung transplants	Immunofluorescence	Ranchoux *et al.* [65]
		Transmission Electron Microscopy	
		Immunoelectron Microscopy	
Cardiac Fibrosis	Heart transplants	Gene Expression	Xu *et al.* [170]
Chronic kidney disease-associated cardiac fibrosis	Heart tissue (Autopsies and cardiac surgery)	Immunohistochemistry	Charytan *et al.* [171]
		Gene Expression	
Diabetic kidney disease-associated renal fibrosis	Kidney biopsies	Immunohistochemistry	Li *et al.* [106]
Idiopathic Portal Hypertension	Liver biopsies	Immunohistochemistry	Kitao *et al.* [62]
Intestinal Fibrosis	Colonic mucosa	Immunohistochemistry	Reider *et al.* [59]
Radiation-induced Rectal Fibrosis	Rectal tissues (Surgery)	Immunofluorescence	Mintet *et al.* [63]
